# SEARCH Study: Text Messages and Automated Phone Reminders for HPV Vaccination in Uganda: Randomized Controlled Trial

**DOI:** 10.2196/63527

**Published:** 2025-05-05

**Authors:** Sabrina B Kitaka, Joseph Rujumba, Sarah K Zalwango, Betsy Pfeffer, Lubega Kizza, Juliane P Nattimba, Ashley B Stephens, Nicolette Nabukeera-Barungi, Chelsea S Wynn, Juliet N Babirye, John Mukisa, Ezekiel Mupere, Melissa S Stockwell

**Affiliations:** 1 Department of Pediatrics and Child Health, School of Medicine, College of Health Sciences, Makerere University, Kampala, Uganda; 2 Kampala Capital City Authority, Kampala, Uganda; 3 School of Public Health, College of Health Sciences, Makerere University, Kampala, Uganda; 4 Division of Child and Adolescent Health, Department of Pediatrics, Vagelos College of Physicians and Surgeons, Columbia University, 617 W 168th Street Suite 115, New York, NY, 10025, United States, 1 2123425732; 5 Department of Immunology and Molecular Biology, Makerere University College of Health Sciences, Kampala, Uganda; 6 Department of Population and Family Health, Mailman School of Public Health, Columbia University, New York, NY, United States

**Keywords:** human papillomavirus, vaccination, adolescent medicine, global health, SMS, mHealth, HPV, vaccine, adolescent, teen, youth, teenager, Uganda, Africa, randomized controlled trial, RCT, caregiver, low income country, middle income country, subSaharan Africa, chi square, regression, mobile health, text messaging

## Abstract

**Background:**

Cervical cancer is currently the leading female cancer in Uganda. Most women are diagnosed with late-stage disease. Human papillomavirus (HPV) vaccination is the single most important primary preventive measure. While research regarding text message vaccine reminder use is strong in the United States, their use has not yet been demonstrated in a preteen and adolescent population in subSaharan Africa or other low- and middle-income countries.

**Objective:**

The objective of this pilot randomized controlled trial was to assess the impact of vaccine reminders with embedded interactive educational information on timeliness of HPV vaccination in Kampala, Uganda.

**Methods:**

In this randomized controlled trial conducted in 2022, caregivers of adolescents needing a first or second HPV vaccine dose were recruited from an adolescent clinic and three community health centres in Kampala, Uganda. Families (n=154) were randomized 1:1 into intervention versus usual care, stratified by dose (ie, initiation, completion) and language (ie, English, Luganda) within each site. Intervention caregivers received a series of automated, personalized text messages or automated phone calls based on family preference. Five messages were sent before the due date, including both static and interactive educational information, with five follow-up messages for those unvaccinated. Receipt of the needed dose by 24 weeks postenrollment was assessed by *χ*^2^, regression, and Kaplan-Meier with log-rank test. All analyses were conducted using intention-to-treat principles.

**Results:**

Overall, 154 caregivers were enrolled (51.3% for dose 1; 48.7% for dose 2) and 64.3% (n=99) spoke Luganda. Among individuals in the intervention arm, 62% (48/78) requested SMS text message reminders and 38% (n=30) requested automated phone reminders. There was no significant difference in requested mode by HPV vaccine dose or language. Intervention adolescents were more likely to receive the needed dose by 24 weeks (51/78, 65.4% vs 27/76, 35.5%; *P*<.001; RR 1.8; 95% CI 1.3‐2.6). There was no interaction by dose or language. There was no difference in vaccination between those requesting SMS text message versus phone reminders (32/49, 65.3% vs 19/30, 63.3%; *P*=.86). The number needed to message for one additional vaccination was 3.4 (95% CI 2.2‐6.8). Kaplan-Meier curves demonstrated more timely vaccination in the intervention arm (*P*<.001).

**Conclusions:**

In this novel trial, SMS text message and automated phone reminders were effective in promoting more timely HPV vaccination in this population.

## Introduction

Worldwide, human papillomavirus (HPV) infection is the most common sexually transmitted infection [1], with the highest prevalence in young adults [2]. The majority of HPV-associated morbidity and mortality is due to cervical cancer, which is the fourth most common cancer in women worldwide, with an estimated 604,127 new cases and 341,831 deaths in 2020 [3,4]. SubSaharan Africa overall, has the highest cervical cancer incidence and mortality rates in the world [4]. Cervical cancer is the leading cancer among women in Uganda [4]. Up to 72% of women are diagnosed with late-stage disease [5], and 80% die within 5 years of diagnosis [6]. The age-standardized cervical cancer incidence rate in Uganda is more than 4 times the global average, and the age-standardized mortality rate is nearly 6 times higher [4].

HPV vaccination is the single most important primary preventive measure for HPV [7-9]. A systematic review of HPV vaccine programs across 69 countries over ten years found estimated maximal reductions of 90% for HPV 6/11/16/18 strain infection (ie, the most common cancer-causing strains), 90% for genital warts, and 45% for low-grade and 85% for high-grade cytological cervical abnormalities [10]. HPV also plays an important role in anal, vulvar, penile, and oropharyngeal cancer [4]. HPV vaccines are most effective when adolescents are vaccinated before HPV exposure. HPV vaccination is currently recommended in Uganda for adolescent girls aged 9‐14 years in a 2-dose series and as a 3-dose series for those aged 15‐26 years.

However, despite high efficacy and a strong safety profile, global HPV vaccination coverage rates remain low compared to other routinely recommended vaccines [11]. Uganda added HPV vaccination to the National Extended Programme on Immunization in November 2015. However, among the adolescents who receive the first dose of the HPV vaccine, many do not receive the second dose they need to complete the series for full protection. Furthermore, among those who do not receive the second dose, many do not receive it in a timely fashion—that is, within the recommended 12 months—resulting in more days at-risk. In Kampala City, only 45%‐62% of girls in the target age range have received two doses of the vaccine [12]. Similarly, many girls (56.7%) had delayed completion of HPV vaccination [13].

Reminders are an evidence-based approach to improve vaccine receipt. In Uganda, the only current reminder available is a paper appointment card, which can easily be misplaced or destroyed. A countrywide evaluation of HPV vaccination in Uganda highlighted the need for caregiver engagement to improve HPV vaccination rates. It was also noted that there was an inadequate follow-up system for second doses [14,15]. Text messaging or SMS text messaging offers low-cost, scalable opportunities to foster vaccination across a population. A Cochrane review found that text messaging, as a single-method reminder, had the largest effect size with high certainty of evidence (risk ratio [RR] 1.29, 95% CI 1.15‐1.44; six studies; 7,772 participants) [16]. Text message reminders for HPV vaccination have shown to be effective in several studies within the United States [17-19]. Such reminders have also been used for pediatric vaccination studies in African countries such as Kenya and Nigeria [20-25], and in three other non-African low- or middle-income countries (LMIC) [26-28], but not for adolescent vaccines including HPV vaccine. Adolescent vaccination patterns, knowledge, and perceptions differ from those for pediatric vaccination, and interventions in high-income versus low- or middle-income countries also differ [29]. Therefore, text message performance cannot be extrapolated from those targeted to parents of young children or to parents in high-income settings.

In this pilot randomized controlled trial, we assessed the impact of vaccine reminders with embedded interactive educational information on timeliness of HPV vaccination in Kampala, Uganda.

## Methods

### Setting, Recruitment, and Informed Consent

This randomized controlled trial RCT (clinicaltrials.gov: NCT05151367; 09/12/2021), funded by the National Institutes of Health (R21CA253604), was conducted in 2022. This study took place at three health centres—Kisenyi, Kawaala, and Kiswa—in Kampala, Uganda, overseen by the Kampala Capital City Authority (KCCA) and located in the suburbs of Kampala City, as well as the Makerere/Mulago/Columbia Adolescent Health Clinic at Mulago National Referral Hospital, the teaching hospital for Makerere University College of Health Sciences. The study health facilities were selected purposively in consultation with KCCA to ensure the inclusion of different city divisions. Each of the study health facilities provides HPV vaccination services to adolescent girls and has affiliated schools, although due to the pandemic, vaccination was not occurring in schools.

Caregivers were eligible to participate if they were (1) the adult parent of an adolescent girl aged 10‐14 years due for a first or second HPV vaccine dose during the study period, (2) resided in Kampala or the surrounding districts, (3) had their usual care at a study site, (4) spoke English or Luganda, and (5) had a cell phone with text messaging capability. Only one child per family was included. Families were recruited by study staff, including village health teams, either through the health facilities or in the community. Informed consent was obtained from a parent or legal guardian for study participation. On enrollment, a demographic survey was completed.

### Randomization

We used central randomization of parent-adolescent dyads with a 1:1 allocation, stratified by dose (ie, initiation, completion), and language (English, Luganda) within each site. The randomization lists, based on permuted blocks with varying block sizes, were prepared within REDCap (Research Electronic Data Capture) electronic data capture tools hosted at Columbia University [30,31]. The statistician and those collecting outcome data remained blinded to arm assignment until analyses were complete.

### Intervention

On enrollment, parents randomized to the intervention were able to select if they wanted messages sent either via SMS text messages or as an automated phone call, as well as if they wanted messages in English or Luganda. Messages included both reminders about the needed dose as well as educational information about the importance of the HPV vaccine and were based on a successful previous study [17], as well as 85 key informant interviews with caregivers (n=30), adolescents (n=30), health care workers (n=11), school (n=10), and government officials (n=4). First, team members identified the key content, timing, and framing for each message based on the interviews. The final draft messages were first designed in English, then translated into Luganda by an independent professional translator, and then reverse-translated by a second independent professional translator, while ensuring proper linguistic and cultural equivalence. The Luganda and English messages were then pretested with 24 parents with small iterative changes.

Five messages before the due date and five follow-up messages were automatically sent centrally using a custom-designed SMS text messaging or phone reminder service. Charges were reversed such that no family was charged for sending or receiving messages. Messages included both static and interactive educational information such as the need for timely receipt of HPV vaccines and differed based on which dose was due. Messages for those who had not yet received any HPV vaccine dose began right after enrollment. For those who had received one dose but had not completed the series, messages began either 4 weeks before the dose was due (ie, for those for whom the dose was not yet due) or immediately (if the dose was overdue). Families who reported that their adolescent would be away at boarding school had their message timeline targeted to the next school break. Messages were personalized with the adolescent’s first name. Parents randomized to usual care received no vaccine reminders and instead received their usual care.

### Measures

#### Outcomes

The primary outcome was the receipt of the next needed HPV vaccination (dose 1 or dose 2) by 24 weeks postenrollment. Practice staff blind to study arm assignment abstracted vaccination data from the HPV vaccine registry within each enrolling site.

#### Analyses

For this feasibility pilot study, with a minimal sample size of 77 in each arm, a hazards ratio of 1.67 with 80% power was observed, allowing for a 5% type I error comparing time to next dose.

To assess the primary outcome, we assessed the difference in proportions using *χ*^2^ tests and relative risk estimated from logistic regression. We also assessed for any interaction effects of doses due or language of messages (English or Luganda). The number needed to message for one additional vaccination was then calculated. In addition, the timeliness of vaccination was assessed using Kaplan-Meier analyses with log rank test. All analyses were intention to treat. All analyses were conducted using STATA (version 14.0 MP; STATACorp). A formal statistical analysis plan was drafted and agreed upon prior to the start of analysis.

### Ethical Considerations

This study was approved by the Institutional Review Boards at Columbia University (AAAT1572) and Makerere University (Mak-SOMREC-2020‐13) as well as by the Uganda National Council for Science and Technology, with written consent for study participation. Informed consent was obtained from a parent or legal guardian for study participation. All data were securely stored in a study-specific database. Those enrolled received Ugandan Shillings (UgX) 15,000 which is equivalent to approximately US $4 as compensation for their time at the initial interview.

## Results

**Figure 1.**
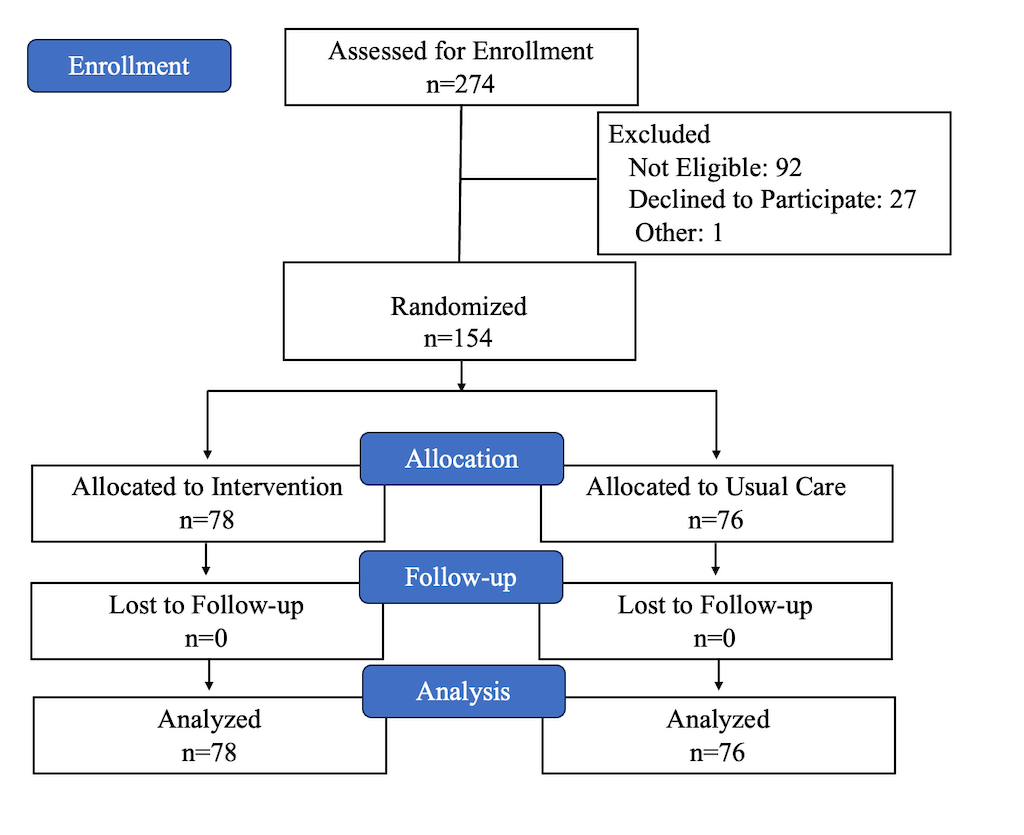
Enrollment flow diagram.

Overall, 274 families were approached and 181 were eligible; of those, 85.1% (n=154/181) were enrolled (Figure 1). Only 8 families were ineligible due to not having a cell phone. Of the 154 dyads enrolled; 51.3% (n=79) had not yet initiated the series and were enrolled to receive dose 1 reminders for initiation; 48.7% (n=75) had already received one dose and were enrolled for dose 2 reminders for completion. No family was ineligible due to not speaking English or Luganda. Nearly two-thirds of those enrolled (64.3%; n=99) spoke Luganda (Table 1), and there were relatively equal numbers enrolled by age group except for a smaller percentage of 14-year-olds. Over three-quarters (77.3%; n=119) were enrolled from the KCCA sites. There was an equal balance between arms for those in the intervention and usual care groups. Of those families randomized to the intervention, 62% (n=48/78) requested the reminders be sent as text messages, with the remaining 38% (n=30) requesting automated phone reminders. There was no significant difference in requested mode based on HPV vaccine dose or language (desired text messages for initiation reminders (22/39, 56%), versus for completion reminders (26/39, 67%; *P*=.35), desired text messages for reminders in English (21/28, 75%) versus in Luganda (27/50, 54%; *P*=.07).

**Table 1.** Characteristics of study participants.

Variables	Total (N=154)n (%)	Intervention (n=78),n (%)	Usual care (n=76),n (%)	*P* value
Previous HPV^a^ doses received				
0 doses	79 (51.3)	39 (50.0)	40 (52.6)	.74
1 dose	75 (48.7)	39 (50.0)	36 (47.4)	
Site				
KCCA^b^	119 (77.3)	61 (78.2)	58 (76.3)	.78
Mulago	35 (22.7)	17 (21.8)	18 (23.7)	
Language				
English	55 (35.7)	28 (35.9)	27 (35.5)	.96
Luganda	99 (64.3)	50 (64.1)	49 (64.5)	
Age of adolescent				
10 years	32 (20.8)	15 (19.2)	17 (22.4)	.60
11 years	38 (24.7)	20 (25.6)	18 (23.7)	
12 years	31 (20.1)	19 (24.4)	12 (15.8)	
13 years	40 (26.0)	17 (21.8)	23 (30.3)	
14 years	13 (8.4)	7 (9.0)	6 (7.9)	
Health status of adolescent				
Fair	7 (4.5)	3 (3.8)	4 (5.3)	.41
Good	32 (20.8)	14 (17.9)	18 (23.7)	
Very good	37 (24.0)	23 (29.5)	14 (18.4)	
Excellent	78 (50.6)_	38 (48.7)	40 (52.6)	
Highest level education caregiver				
No schooling	20 (13.2)	11 (14.1)	9 (12.2)	.83
Primary or O level	102 (67.1)	53 (67.9)	49 (66.2)	
A level or higher	30 (19.7)	14 (17.9)	16 (21.6)	
Sex of caregiver				
Female	130 (84.4)	66 (84.6)	64 (84.2)	.95
Male	24 (15.6)	12 (15.4)	12 (15.8)	

a HPV: human papillomavirus.

b KCCA: Kampala Capital City Authority.

### Characteristics of the Study Population

Most (130/154, 84.4%) of the caregivers were women; 13.2% (20/152) had no schooling, and 67.1% (102/152) had only completed a primary or ordinary level of education. Approximately 54.9% (84/153) had a regular (ie, not smart) cell phone. Of those with a smartphone, none preferred WhatsApp over text messaging to receive health reminders for their adolescent. Of all those surveyed, while most (136/152, 89.5%) were not worried at all about going over their text message limit, 76.6% (118/154) had run out of airtime or messages in the past 6 months. Most (125/154, 81.2%) received text messages daily. Nearly half (76/154, 49.4%) reported receiving most of their information about the HPV vaccine from a health care provider, 27.9% (n=43) from radio or television, 7.1% (n=11) from family or friends, and 2.6% (n=4) at their adolescent’s school. Approximately 41.6% (64/154) knew their adolescent was due for an HPV vaccine dose, and of those 46.9% (30/64) knew when the dose was due. The HPV vaccine perceptions were generally positive, with 96.7% (147/152) reporting that the vaccine was very or somewhat safe, and 98.7% (151/153) reporting that it worked very or somewhat well. Only 44.4% (68/153) thought their adolescent would be protected with only one dose of the vaccine.

### Intervention Effects

**Figure 2.**
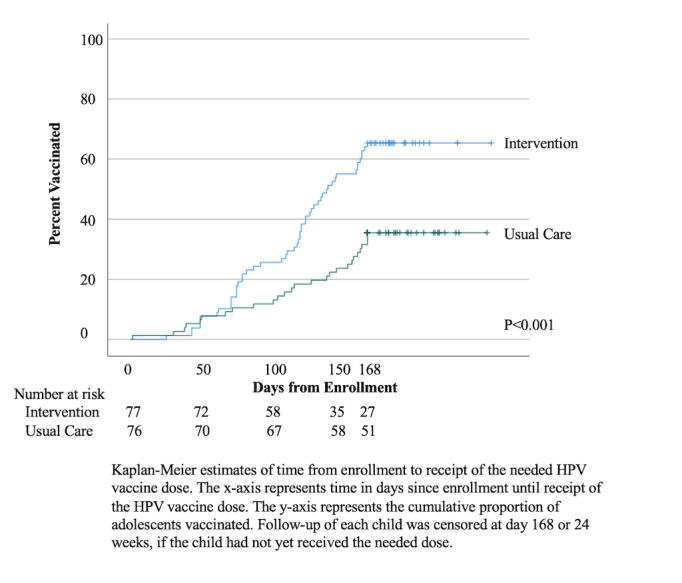
Time from first message sent to receipt of next needed dose.

Overall, 95.5% (703/736) of text messages were delivered as expected and 90% (9/10) of replies were processed as expected. In an intention-to-treat analysis, intervention adolescents were more likely to receive a needed dose by 24 weeks (65.4%, 51/78 vs 35.5%, 27/76; *P*<.001; RR 1.9 95% CI 1.3‐2.6). As an exploratory analysis, there was no interaction by dose needed (*P*=.34) or language (*P*=.22). There was also no difference in vaccination between those who wanted the reminders sent as a text message or as an automated phone reminder (32/49, 65.3% vs 19/30, 63.3%; *P*=.86). The number of families needed to message for one additional girl to be vaccinated was 3.4 (95% CI 2.2‐6.8). Kaplan-Meier curves demonstrated more timely vaccination (*P*<.001) (Figure 2). On post-hoc analysis, when split by dose, results were similar; the number of individuals who needed dose by 24 weeks for initiation was 66.7% (26/39) versus 27.5% (11/40; *P*<.001; RR 2.17 95% CI 1.34‐3.52) and for completion was (25/39, 64.1% vs 16/36, 44.4%; *P*=.09; RR 1.54 95% CI 0.93‐2.58).

### Postintervention Interviews

We completed postintervention interviews with 30 parents from the intervention arm, including those in need of first versus second doses, those who requested text versus phone reminders, and those who spoke English versus Luganda. Overall, all study participants rated the reminders as highly acceptable and would recommend them to their friends. Some viewed the reminders as a demonstration of “special care” by health workers to have adolescent girls vaccinated and protected against cervical cancer. Participants noted the messages reminded them of when to take their girls for HPV vaccination and provided additional information on the safety and importance of HPV vaccine. A few participants expressed an inability to read messages on their own, receiving few messages, and having busy schedules—highlighting the continued importance of offering an option for automated phone reminders instead of solely text messages. All participants agreed that the intervention should be extended to other community members.

## Discussion

This pilot trial demonstrated that SMS text message and automated phone vaccine reminders for HPV vaccination were feasible and acceptable for parents to use in a low-income country such as Uganda, with high user satisfaction. This pilot study also demonstrated effectiveness in a combined outcome of next needed dose—first or second dose—in both overall vaccination at 24 weeks as well as timeliness of receipt of the needed dose. The overall number of families needed to message for one additional girl to be vaccinated was quite low at 3. Overall, our data suggest that integration of SMS text message vaccine reminders in LMICs is both feasible and accepted by parents and could improve HPV vaccination across a population.

Reminder strategies may be especially important for adolescents, who unlike young children in general have fewer visits with health care providers and therefore, may be less likely to be vaccinated or receive information about the vaccine. In a study conducted in Australia, SMS reminders to parents or guardians who had consented to their adolescent receiving the HPV vaccine within a school-based program led to higher HPV vaccine administration [32]. Similarly, text message reminders for HPV vaccination have been shown to be effective in several studies within the United States [17-19]. In our study, parents in the intervention arm received personalized, scheduled message reminders that included both a reminder about the needed dose as well as educational information about the importance of the HPV vaccine. This addition of information likely proved important, given that only half of the participants knew their adolescent was due for an HPV vaccine, and of those who did, only half of the participants knew when the dose was due. The countrywide evaluation of HPV vaccination in Uganda highlighted the need to increase awareness of the importance of HPV vaccination, as well as evidence-based education and outreach [14,15]. Perceived vaccine safety [5] and perceived benefits of vaccination—particularly cancer protection [33]—have all been noted before as important factors that may impact HPV vaccination in Uganda, all of which were included in our messages.

Some important lessons were learned in this pilot study that could be important for others wanting to use text message vaccine reminders. First, there was high user satisfaction, acceptability, and recommendation ratings, illustrating general interest in these sorts of reminders. Second, only 8 families were ineligible due to not having a cell phone, indicating the potential reach of such interventions. The Global System for Mobile Communications Association Intelligence data highlight that there were 30.55 million cellular mobile connections in Uganda at the start of 2023, which is equivalent to 63.8% of the total population [34]. However, other LMIC may have less cell phone penetration. Third, although a minority, some families opted to receive automated phone reminders, highlighting the potential importance of families being able to select their mode of reminder. There was also no effect on the intervention based on type of reminder selected. In a study of HPV vaccine reminders in the United States, text messages but not phone reminders were effective [18]. It is not clear if the difference here is that families had a choice in their reminder types. Another study, also in the United States but focusing on HPV vaccine series completion, had families choose the modality of the intervention and found completion rates were similar between different recall methods [35]. Of note, only half of the participants in our study had a smartphone, highlighting the continued importance of SMS versus multimedia messaging service or web-linked text messages for this population. Fourth, while most caregivers reported that they were not worried at all about going over their text message limit in a month, three-quarters of them reported that they did run out of airtime or messages in the past 6 months. In this study, we set up reverse billing. It is not clear if the parents would have opted to pay for their own messages. If such reminders were to be used across a larger population for future vaccination support, reverse billing may be needed. Of note, in previous studies in the United States, cost of messages was not highlighted as a concern [36]. Finally, we needed a long lead time to collaborate with a vendor and receive a code from the Uganda Communication Commission; therefore, others may need to keep a long lead time in mind. While not text message reminder-related, it may be important to note that only 44.4% of caregivers surveyed thought their adolescent would be very protected with only one dose of the vaccine. If Uganda moves to a one-dose HPV vaccine, as may be recommended [37], much education may be needed to reassure families that only one dose is now needed.

This study had several limitations. First, our participants were a convenience sample who agreed to take part in an HPV vaccine text messaging study and were from an urban and primarily literate population and therefore may not be representative of the larger population. Similarly, HPV vaccine perceptions regarding safety and efficacy were generally positive among participants. It is unclear if, among a population with more negative views, the intervention would be as successful. However, despite the positive views only one-third of the usual care group were vaccinated within the 24-week period. Third, not 100% of the text messages were delivered as expected. Since all analyses were intention-to-treat, this could have impacted our effect size. We were also not able to confirm delivery of automated phone calls. Fourth, we allowed participants to select text message versus automated phone reminders and therefore could not assess impact if we had not allowed selection and instead sent all participants text messages. Next, this trial took place during the COVID-19 pandemic which could have affected vaccine receipt. However, this should have affected both arms equally. Finally, this pilot study was designed for a combined outcome of next needed dose—first or second dose. Future work will involve conducting a larger, fully powered randomized control study. Despite these limitations, a key strength of this study is that it demonstrated feasibility, acceptability, and pilot effectiveness in over 150 families, recruited from different areas in Kampala, and included English and Luganda speakers. Luganda is one of the most spoken languages in Uganda.

In this novel trial, text message and automated phone vaccine reminders were effective in promoting more timely HPV vaccination. Such reminders could be considered to increase vaccination rates across a population.

## Supplementary material

10.2196/63527 Checklist 1 CONSORT-eHEALTH checklist (V 1.6.1)

## Abbreviations

HPV: Human papillomavirus
KCCA: Kampala Capital City Authority
LMIC: Low- or middle- income countries
REDCap: Research Electronic Data Capture
UgX: Ugandan Shillings
